# Reply to Aguenaou et al. Comment on “Muzzioli et al. Are Front-of-Pack Labels a Health Policy Tool? *Nutrients* 2022, *14*, 771”

**DOI:** 10.3390/nu14102167

**Published:** 2022-05-23

**Authors:** Luca Muzzioli, Claudia Penzavecchia, Lorenzo Maria Donini, Alessandro Pinto

**Affiliations:** Department of Experimental Medicine, Sapienza University, 00185 Rome, Italy; muzzioli.680093@studenti.uniroma1.it (L.M.); c.penzavecchia2@gmail.com (C.P.); lorenzomaria.donini@uniroma1.it (L.M.D.)

As a team of scientists who believe that exchanging views is one of the prerequisites of the scientific method, we welcome the comments from Aguenaou H et al. [1], and we would like to clarify some aspects that appear to be misinterpreted.

In line with the introductory paragraph of our paper [2], FOPLs are considered tools “to help consumers make the right choices from a nutritional standpoint when buying food products”. The aspect we caution about, as the main reason underpinning our review, is that “the FOPL system must be adequately tested and validated, and, once adopted, it must lead over time to a real and significant improvement of health status in the European Community” [2].

Moving on to the individual criticisms made by Aguenaou et al. [1], the following aspects should be noted:

1. We do not place informative and interpretive labels at the same level. In fact, we underline that informative and interpretive labels “have been developed with different purposes, but they are usually compared to identify the most effective one, so they either fail or succeed depending on the primary objective of the study. They should be perceived as two sides of the same coin rather than as competitors” [2];
2. The document published by the WHO [3] and cited by Aguenaou H et al. [1] highlights that “emerging findings of primary research studies, […] provide new evidence on the potential effectiveness of other types of systems, […] (and) the generalizability of research findings on a FOPL system’s performance across countries may be variable, given differences in prior exposures. It is prudent for countries to undertake consumer testing of proposed FOPL systems to ensure their suitability for the target market”. Moreover, on 27 September 2021, the WHO stated that “at present WHO is not able to recommend the use of any specific scheme over another” [4];
3. Regarding the use of FOPLs as drivers for reformulation, data reveal that “studies on food manufacturers’ responses to FOP labels are limited in both number and strength of evidence”, as stated in the document published by the European Commission in 2020 [5]. Even in the study proposed by Aguenaou et al. [1], the authors stated that “reformulation changes following voluntary Health Star Rating (HSR) labelling are small, but greater for initially unhealthy products. Initially unhealthy foods were, however, less likely to adopt HSR” [6];
4. In the paper by Grunert et al. [7], theorised as a study framework, there is also a call for an urgent “need (for) more insight into whether labels actually are used in guiding buying decisions and with what effect”, confirming the lack of a consensus in the scientific community. Moreover, the authors stated that “this is a difficult topic and can only be investigated by using a combination of different approaches” to research how FOPLs affect consumers’ dietary patterns and their behaviour in real-world setting, which is precisely what we suggested and that “there is, however, virtually no insight into how labelling information is, or will be, used in a real-world shopping situation, and how it will affect consumers’ dietary patterns”;
5. The meta-analysis by Song et al. [8] had not yet been published at the time of our literature review. Nevertheless, even if the authors found that different labels appear to have beneficial effects on the purchase of healthy food products, they also stated that “study limitations included high heterogeneity and inconsistency in the comparisons across different label types, limited number of real-world studies (95% were laboratory studies), and lack of long-term impact assessment”. Moreover, the authors acknowledged a certain number of limitations: “first, compared to the relatively large amount of evidence on the purchasing behaviour elicited by FOPLs, the data on food consumption were quite limited. […] However, the research gap between purchase and actual intake of different nutrients remains to be validated by future studies, which is crucial to inform decision-making on labelling policies. Second, most of the studies were laboratory experiments […]. There were very few real-world studies assessing the effect of mandatory labelling policies on genuine purchase. […] Third, more than half of the studies included had a high risk of bias”;
6. The papers considered by Aguenaou et al. [1] to “help consumers identify healthier foods and make healthier purchases and contribute to reduce the burden of nutrition-related diseases” [9,10] are retrospective studies in which the spontaneous (not FOPL-driven) consumption of healthy foods was estimated to be positively associated, a posteriori, with lower risk of obesity and/or non-communicable diseases. In fact, the studies have not verified the real impact of the food certification system on the health status, but they only assessed the risk of disease through a macro-simulation;
7. Concerning our citation of the study by Hagmann et al. [11], we confirm that the results of the study show that “the Nutri-Score label was perceived as the least useful type of nutrition information in the overall sample” (see Section 3.4);
8. It would be advisable for the authors’ last peremptory statement to be less trenchant given that, despite the study they cite [12], WHO states that the “WHO encourages Member States and research institutions to continue analysing information, with the purpose of better understanding the impact of different FOPL schemes in different contexts” [4];
9. We maintained a balanced approach to the FOPL question underlining that FOPLs must be framed by taking a broader approach, where different food categories, different countries with their guidelines, different food cultures, and different grades of exposition to the labels are taken into account in the studies in agreement with the World Health Organization Guiding Principles. We believe that it is not correct to interpret our revision as an “overstating of the merits of non-interpretive labels”.

Finally, due to our lack of any conflicts of interest in either interpretive or informative labels, the aim of our narrative review was to provide useful instruments to encourage the readers in critical thinking towards the subject and to pose some doubts as a scientific approach to gain knowledge.

The term “unequivocally” is not part of our vocabulary as researchers.
